# Prioritizing the Journey and the Destination: Comment on "Achieving Diagnostic Excellence: Roadmaps to Develop and Use PatientReported Measures With an Equity Lens"

**DOI:** 10.34172/ijhpm.8907

**Published:** 2025-03-11

**Authors:** Theresa Schmidt, Jacqlyn Riposo

**Affiliations:** Independent Researcher, Alexandria, VA, USA.

**Keywords:** Patient-Reported Outcomes, Quality Measures, Patient-Centered Care, Diagnostics, United States

## Abstract

McDonald and colleagues’ paper on "Achieving Diagnostic Excellence: Roadmaps to Develop and Use Patient-Reported Measures With an Equity Lens" describes seven goals for using patient-reported measures (PRMs) to promote diagnostic excellence and describes roadmaps to plan the development and implementation of PRMs. Incorporating more patient voices into diagnostic measurement and measure development can improve the patient-centricity of processes and outcomes. Additionally, organizations beginning their measure development journeys may find road-mapping tools helpful, especially the focus on setting goals and engaging stakeholders. However, the authors do not offer suggestions for prioritizing measure concepts for development, and the long timeframes of the examples may dissuade some organizations from engaging in measurement to begin with. Real-world examples of measure development processes and potential applications of emerging technologies are important complements to aspirational roadmap goals and processes.

## Introduction

 Patient-reported data is considered a “gold standard for understanding the impact of the diagnostic process on patients.”^1^ McDonald and colleagues’ article^2^ argues that an essential component of measuring diagnostic excellence is incorporating patient-reported measures (PRMs) into measurement strategies and introduces roadmaps as tools to support multistakeholder development of PRMs. While McDonald and colleagues approach the topics of diagnostic excellence goals, PRMs, and road-mapping from a global perspective, this commentary will focus on the utility of the road-mapping technique and discuss potential real-world applications in the United States.

## Importance of Patient-Reported Measures in Value-Based Care

 McDonald’s and colleagues’ vision of a diagnostic excellence measurement system that centers patient and family voices is laudable. As the authors correctly assert, PRMs are the most appropriate way of capturing concepts for which patients are the best source of information. The authors highlight some of the most relevant elements of the diagnostic process where this is the case (eg, patient understanding of diagnosis, patient activation) and how PRMs might be used to support diagnostic excellence (eg, via organizational quality improvement activities).

 The Expert Convening process and published output are excellent examples of embedding patients and patient advocates in various roles throughout the project: advisors, members of the research team, and convening participants. By including as equal partners in decision-making the people with the most experience in patient experience—patients themselves—the researchers sought to expand the definition of diagnostic excellence to better reflect the values of patients.

## Relative Value of the Diagnostic Goals

 The authors’ vision for using PRMs to support diagnostic excellence across seven identified goals is aspirational. While the authors make a case for the value of using PRMs in each of the seven areas, they do not put more weight on one area of excellence over another or prioritize specific measure concepts for development, perhaps because the heterogeneity of national healthcare systems across the globe results in different needs that PRMs can fill.

 We and others have written about the challenges in implementing PRMs in the United States, including patient and provider burdens as well as interoperability.^3^ Given these considerations, along with the lengthy proposed timelines for rigorous, equitable, and multistakeholder development of meaningful and useful PRMs, we suggest prioritizing cross-cutting concepts that meet several criteria:

1. Are meaningful to patients across care settings. Measures that can capture patient experiences in the hospital, outpatient, post-acute, and other settings might not only reduce implementation burden, but could facilitate comparisons between different provider types and track patient and system progress over time.
2. Contribute to clinical care, quality improvement, and value-based payment. A PRM that captures patient understanding of diagnosis, for example, could identify patients in need of further education, assess provider or payer communication improvement over time, and/or be implemented in a value-based payment program.
3. Cannot be measured in other ways. Concepts that reflect patient and family perspectives or lived experience are often best or exclusively captured via PRMs.

The article’s proposed “equitable patient-centeredness” composite is an example of a measure that could meet all three of these criteria. Other examples include concepts like health-related quality of life, understanding of diagnosis (noted above), feeling “heard and understood,”^4^ experience of racism or discrimination, shared decision-making, and goal-concordant care (a holy-grail concept that has eluded measure developers). These examples are relevant to multiple settings, best reported by patients, and can support both diagnostic excellence and treatment quality.

 In addition to the three criteria outlined above, initial exploration of potential measure concepts for development should pursue assessments to determine whether the measures would be important to patients and families, be feasible for implementation from a cost and burden standpoint, be associated with a current gap in care (including disparities in care), and have the potential for improvement.^5^

## Utility of the Road-Mapping Technique

 Beyond the diagnostic excellence goals, the road-mapping technique itself presents utility for organizations seeking to develop and implement PRMs of diagnostic excellence. Each roadmap is oriented around a goal for implementing a PRM, which becomes the terminus of the steps in the roadmap. These steps move from measure identification and development, to endorsing measures, to implementation, and then to practice improvement, finally resulting in impact. The roadmap notes synergies and challenges that can either support or undermine the process steps. The roadmap also includes timelines for each step.

 The road-mapping technique may be useful for organizations trying to think through their strategies for pursuing PRM development related to diagnostic excellence or any other topic. Perhaps the most important contribution here is the idea of starting with the end in mind - what is the organization trying to accomplish via measurement? By focusing in on measurement goals, vs. prescribing the specific PRM to be developed, developers can identify the steps needed to reach those goals along with the challenges to mitigate and synergies to leverage to promote success. In the example roadmap described in the main paper, the goal “Reduced Preventable Care Utilisation Due to Rapid Diagnostic Excellence Patient-Reported Measure Alerts” directly connects the use case for the PRM being assessed (the diagnostic PRM alerts) to a measurable desired outcome. Three other roadmaps are presented in the supplementary materials with goals of “improvement in diagnostically salient communication,” “examination of reasons and types of distrust among those not engaged with the health system in their diagnostic capacities,” and creating “geographic maps of diagnostic excellence disparities.” Only one of these goals (diagnostically salient communication improvement) seems likely to be measured as improvement in performance of the PRM itself. All goals, however, are indicators of successful PRM implementation.

 Once a goal has been defined, the organization can then develop realistic timelines that include not just the development and implementation processes, but also using data from the measures to drive improvement. The timelines in the example roadmaps may be realistic from the standpoint of the experts that created them, especially if those experts are considering using the rigorous Centers for Medicare & Medicaid Services’ (CMS) blueprint process in the context of the US healthcare system. However, they may also mislead the casual reader. In the patient-reported alerts example, the measure development phase takes place over 4 years, and reaching the goal is not expected until year 15. The authors note that the timeline includes multiple feedback loops, “implies continuous learning cycles,” and “assumes no coordinated effort.” Some of McDonald and colleagues’ example roadmaps are shorter (eg, the roadmap towards improvement in diagnostically salient communication timeline is 8 years), and their caveats suggest that even shorter timelines may be possible. This is an important point to emphasize because the timelines as presented may dissuade some organizations from beginning measurement activities in fear that they will not be able to benefit for nearly a decade.

 While the road-mapping technique is useful in providing a standardized process that can be adopted internationally, the timeline and end goal for a given measurement initiative will be very different depending on the payment and incentives structure of each national healthcare system. For example, as the US healthcare system moves from fee-for-service towards value-based care, the incentives structure for value-based payment programs may motivate measure uptake to move more quickly.

 The authors also emphasize the importance of engaging a broad group of stakeholders early and often throughout the roadmap process. A supplementary figure names thirteen different stakeholder types, from patients and providers to government and digital health professionals. The human-centered design principles deployed in this study could potentially support engagement across these stakeholder groups. Engagement strategies from other measurement initiatives may be leveraged to support both road-mapping and measurement development activities. For example, the CMS blueprint recommends engaging technical expert panels that include a variety of stakeholders (eg, patients and families, specialty societies), and organizations like CMS and PFCCpartners have developed guiding principles for engaging patients and families in measure development.^5-7^ The authors also suggest that synchronizing PRM efforts for diagnostic excellence could benefit from a “coordinating centre,” but they do not describe what type of organization might run or fund such an entity.

 Similarly, McDonald and colleagues do not attempt to describe how to implement the roadmaps in a given context or how to execute the steps included in the roadmap, but other resources may be instructive. We have long considered the CMS measures blueprint as a “bible” for the development of measures that are appropriate for use in value-based programs in the United States.^4^ CMS has noted that their measures’ lifecycle also applies to PRMs and has issued specific guidance for developing these measures.^8^ While CMS resources are tailored for a very specific measurement use-case, most of the tools and techniques can be applied to development of measures for other purposes. An early step in the measure lifecycle is conducting an environmental scan that includes reviewing published literature and guidelines, searching for existing measures, talking with experts, and other techniques to assess the landscape related to a given measure concept. This step is critical because it can identify synergies and promote coordinated efforts, both tactics that can shorten measure development timelines.

## Applying Road-Mapping to Real-World Measure Development

 To see how the roadmap timeframes and stakeholder engagement might align with real development experiences, we selected a relevant measure development project as an example. The “Gains in Patient Activation Measure (PAM)” illustrates a measurement process that reaps the benefits of measurement much earlier and in a different order than the prototypical roadmap cycle as described. PAM was first developed in 2004 to capture “the broad range of elements involved in activation, including the knowledge, skills, beliefs, and behaviors that a patient needs to manage a chronic illness.” The concept development and refinement process relied heavily on engagement with a national expert consensus panel and patient focus groups.^9^ Although the measure did not have uptake in national CMS programs until recently, results from clinical practice implementation became available as early as 2007.^10^ In 2016, the measure was endorsed by the National Quality Forum.^11^ It was finalized through rulemaking in the Merit-Based Incentive Payment System program for implementation in 2024 and included in many of the Merit-Based Incentive Payment System Value Pathways.^12^ Even though the path to widespread uptake in national programs was a 20-year journey, the measure began to impact patient care just a few years after it was developed.

## Leveraging Artificial Intelligence to Democratize Development and Reduce Timeframes

 As McDonald and colleagues note, artificial intelligence (AI) can support data collection and analysis of PRMs. AI has already been deployed in measure development and testing in the diagnostic space.^1^ AI-driven natural language processing has also been used to identify potential patient-reported outcomes from social media posts.^13^ We envision a future where AI will make measure development processes quicker, more efficient, and more accessible. By augmenting human intelligence to streamline development processes and shortcut roadmaps, emerging technologies can democratize access to measure development and allow organizations to focus on improving performance on meaningful measures.

## Conclusions

 We applaud McDonald and colleagues’ efforts to promote diagnostic excellence and describe roadmaps to plan the development and implementation of PRMs. We explored potential applications of how road-mapping could be implemented in the US and discussed the real-world example of PAM as a PRM that demonstrated benefits of measurement much earlier and in a different order than the roadmap framework would suggest. When thinking about the application in the US healthcare system, we encourage measure developers to begin PRM development by (1) prioritizing goals of the PRM, (2) identifying efficiencies in the road-mapping framework to reduce the overall timeline, and (3) considering the application of AI and other emerging technologies to find these efficiencies and shift focus toward what is important: advancing excellence and equity in diagnostic outcomes and experiences.

## Ethical issues

 Not applicable.

## Conflicts of interest

 TS and JR are employees of Real Chemistry, a consulting firm with many clients including government, life sciences, non-profit, and provider organizations.
